# Supplementary material for: Biocompatible, Multi-Mode, Fluorescent, T2 MRI Contrast Magnetoelectric-Silica Nanoparticles (MagSiNs), for On-Demand Doxorubicin Delivery to Metastatic Cancer Cells
Source: Pharmaceuticals (Basel). 2022 Sep 30;15(10):1216. doi: 10.3390/ph15101216 (PMC9607636; doi:10.3390/ph15101216)
Supplement: Supplementary file 1 [file pharmaceuticals-15-01216-s001.zip › pharmaceuticals-1930291-supplementary.pdf]

Supplementary-Information:

Biocompatible, Multi-Mode, Fluorescent, T2 MRI Contrast Magnetoelectric-Silica Nanoparticles (MagSiNs), for On Demand Doxorubicin Delivery to Metastatic Cancer Cells.

Margo Waters [1,2†], Juliane Hopf [2,†], Emma Tam [2,3], Stephanie Wallace [2,4], Jordan Chang [1,2], Zach Bennett [2,5], Hadrian Aquino [6], Ryan K. Roeder [7], Paul Helquist [8], M. Sharon Stack [2,9] and Prakash D. Nallathamby [2,7,9,*]

[1] Dept. of Pre-Professional Studies, University of Notre Dame, Notre Dame, IN, 46556 USA;
[2] The Berthiaume Institute for Precision Health, University of Notre Dame, Notre Dame, IN, 46556 USA;
[3] Department of Art, Art History & Design, University of Notre Dame, Notre Dame, IN, 46556 USA
[4] Department of Mathematics and Pre-Professional Studies, University of Notre Dame, Notre Dame, IN, 46556 USA
[5] Department of Aerospace and Mechanical Engineering, University of Notre Dame, Notre Dame, IN, 46556 USA
[6] Electrical Engineering, University of Notre Dame, Notre Dame, IN,46556 USA;
[7] Bioengineering Program in the Department of Aerospace and Mechanical Engineering, University of Notre Dame, Notre Dame, IN, 46556 USA;
[8] Department of Chemistry and Biochemistry, University of Notre Dame, Notre Dame, IN, 46556 USA;
[9] Harper Cancer Research Institute, University of Notre Dame, Notre Dame, IN,46556 USA
* Correspondence: pnallath@nd.edu; Tel.: +1-(574)-631-7868
† These authors contributed equally to this work.

Keywords: magnetoelectric; T2-contrast; fluorescent nanoparticle; cobalt ferrite; silica shell; ON-Demand drug delivery; stimuli responsive drug delivery; doxorubicin; off-target toxicity

Figure S 1: Magnetoelectric nanoparticles synthesized according to existing protocols in peer-reviewed literature [Rodzinski, A.; Guduru, R.; Liang, P.; Hadjikhani, A.; Stewart, T.; Stimphil, E.; Runowicz, C.; Cote, R.; Altman, N.; Datar, R.; Khizroev, S., Targeted and controlled anticancer drug delivery and release with magnetoelectric nanoparticles. *Sci Rep* **2016,** *6*, 20867]

A.

B.

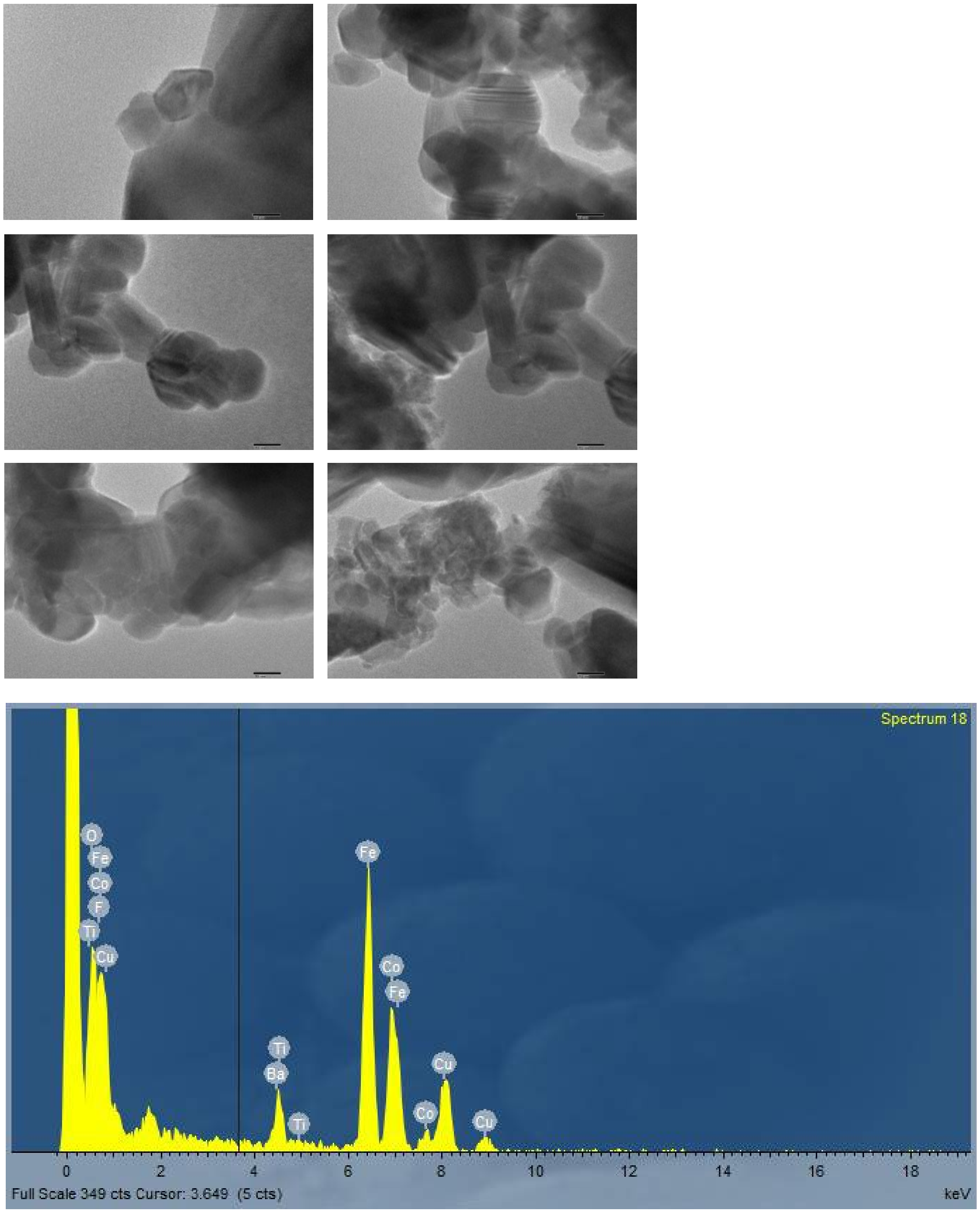

(A) Magnetoelectric nanoparticles with a cobalt ferrite core and a barium titanate piezoelectric shell had net diameters larger than 40 nm. It was quite hard to control the thickness of the barium titanate shell consistently. **(B)** EDXS was used to confirm the elemental composition of the MENs magnetoelectric nanoparticles.

Figures S2: Fluorescence Calibration curve used to estimate FITC release from MagSiNs

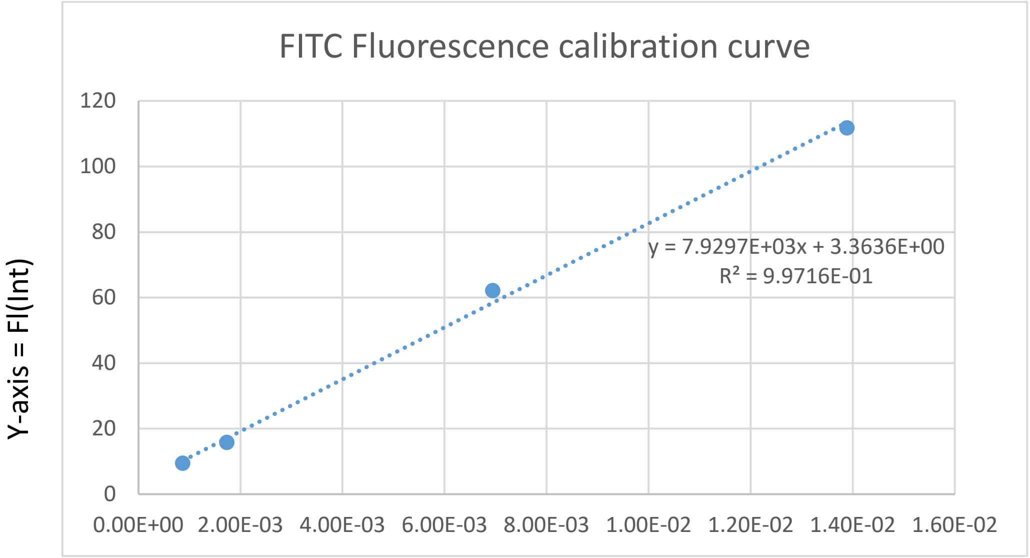

X- axis = mg/mL of Fluorophore

- Excitation wavelength (nm) 494;
- Emission wavelength (nm) 518
- Ex slit (nm) 2.5 nm; Em slit (nm) 2.5 nm

Figures S3: Different modes of Drug Loading and Release

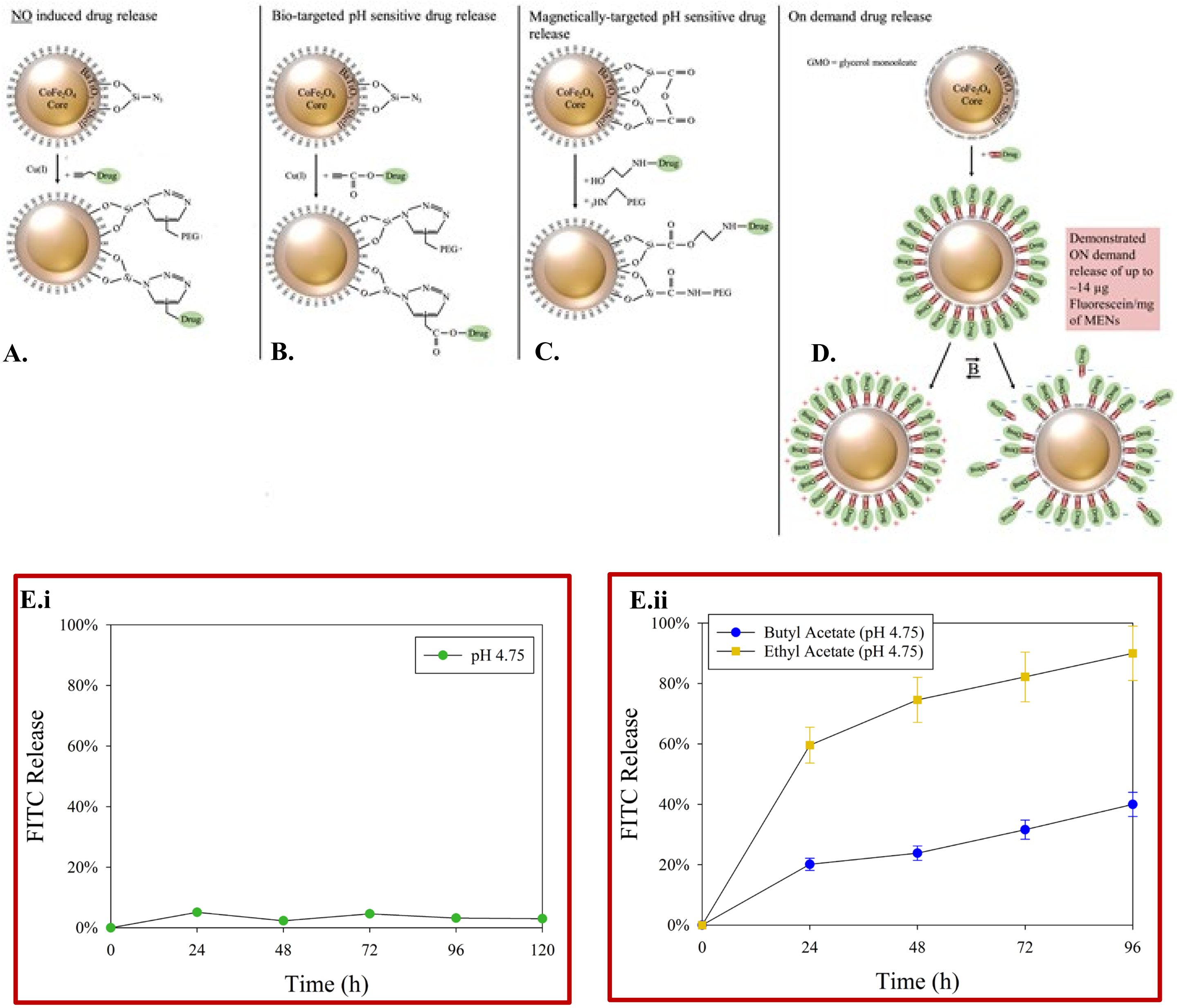

Listed are the other modes of drug delivery that were explored. **(A)** Using CLICK chemistry to link the drug molecule covalently to the MagSiNs to see if the drug was still therapeutically active while conjugated to the MagSiNs surface. **(B)** Using acid-labile ester linkers to release the drug payload from MagSiNs. An additional antibody for increased targeting to cancer cells was also conjugated to the MagSiNs. **(C)** Using acid-labile ester linkers to release the drug payload from MagSiNs. Magnetic field was used for targeting MagSiNs to target cells. **(D)** Electrostatic loading of drugs on MagSiNs followed by electromagnetic release of the drug payload.

(E.i) The high stability of covalently bound FITC to MagSiNs surface at acidic pH 4.75, over 5-days at 37˚C and 5% CO2. **(E.ii)** The release rate of FITC bound to MagSiNs using acid-labile easter linkers as a function of the length of the carbon spacer (2-carbon or 4-carbon) linking the FITC to the ester functional group at acidic pH 4.75, over 4-days at 37˚C and 5% CO_2.

Figure S4: Electromagnet array used for exposing cells to unidirectional magnetic field by passing a DC current or an alternating magnetic field by passing an AC current through the electromagnet array.

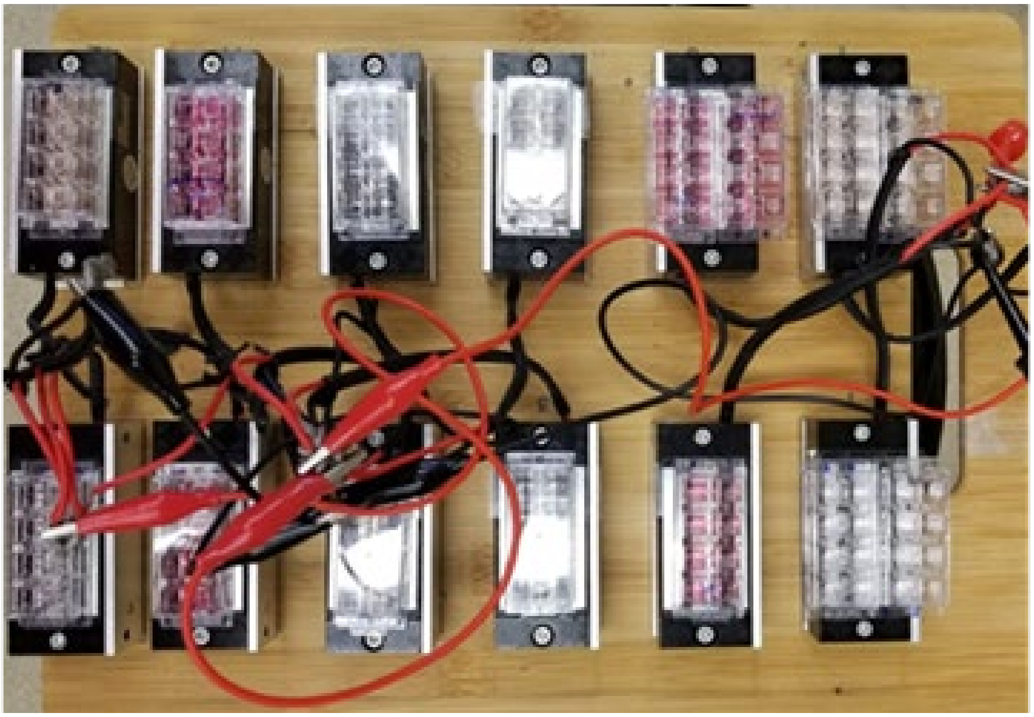

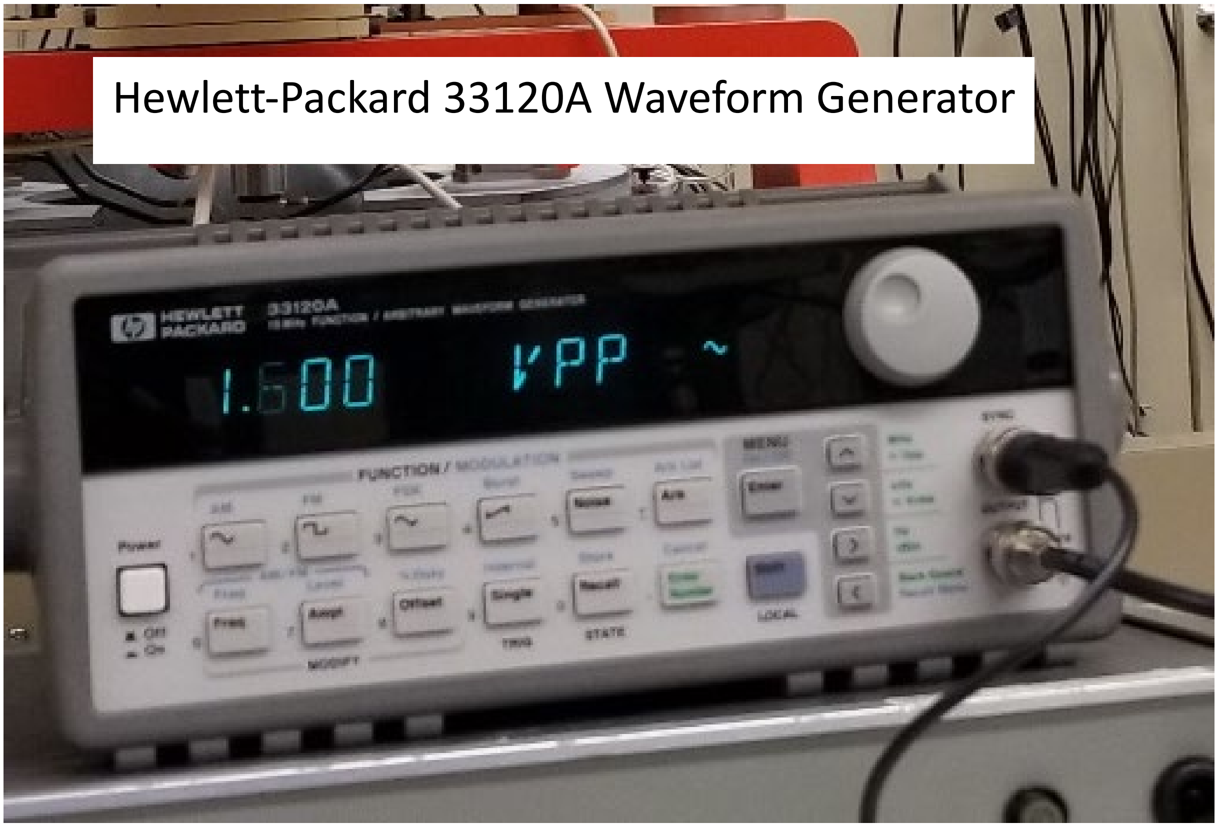

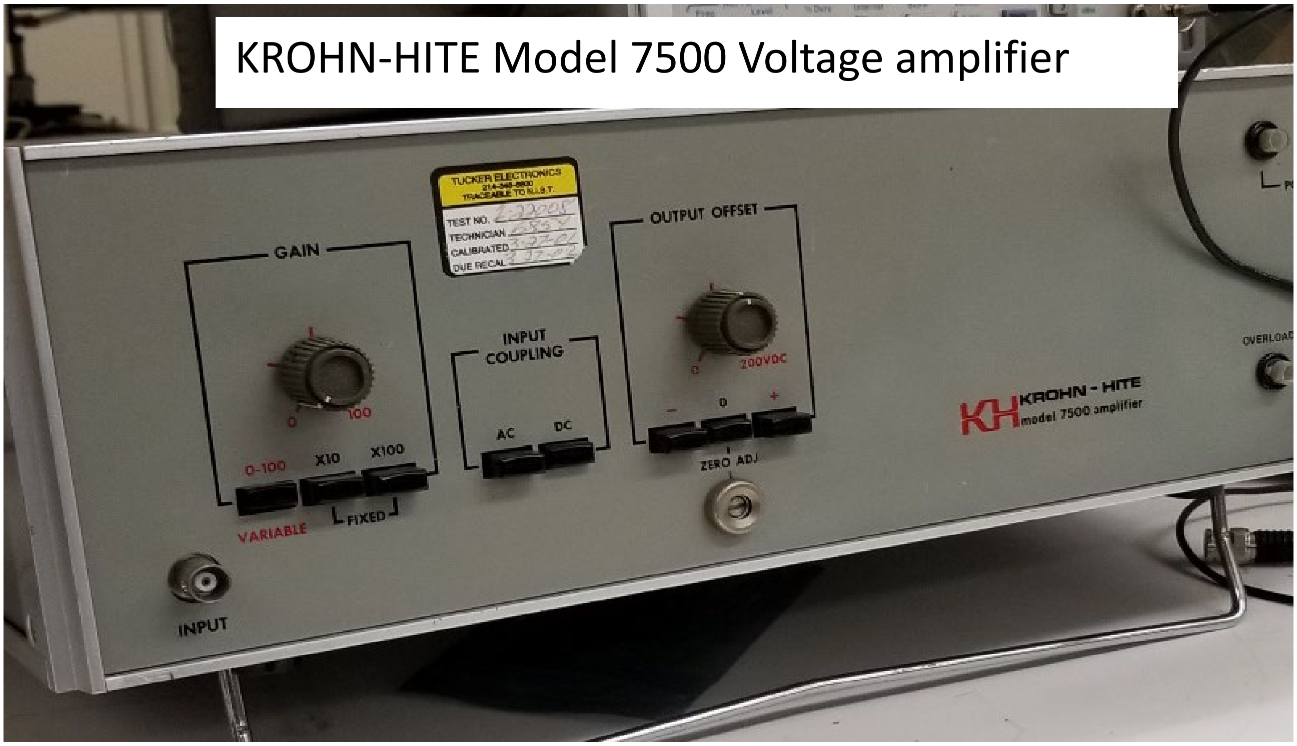

KROHN-HITE Model 7500 Voltage amplifier

The electromagnet array was connected in series. A waveform generator (Hewlett-Packard 33120A) was used to deliver the appropriate signal waveform to a voltage amplifier (KROHN-HITE Model 7500). The amplified signal was then connected to the electromagnetic array. A Gauss meter was used to ensure that the magnetic field strength at a 1cm height above the electromagnets was between 23 Gauss to 40 Gauss. Each electromagnet received an average voltage of 2.4V to 3V.
